# A Route Less Traveled: Anomalous Venous Drainage of the Right Heart

**DOI:** 10.7759/cureus.3339

**Published:** 2018-09-20

**Authors:** Brian Sifrig, Taylor S Harmon, Erik Soule, Joanna Kee-Sampson, Mario Agrait, Todd Loper, Jerry Matteo

**Affiliations:** 1 Radiology, University of Florida College of Medicine, Jacksonville, USA; 2 Interventional Radiology, The University of Texas Medical Branch, Galveston, USA; 3 Interventional Radiology, University of Florida College of Medicine, Jacksonville, USA; 4 Interventional Radiology, Flagler Hospital, St. Augustine, USA

**Keywords:** venovenous anomaly, small cardiac vein, svc syndrome, occlusion, coronary sinus, congenital anomaly, central venous catheter, esrd (end stage renal disease), esrd (end stage renal disease), per cutaneous coronary intervention

## Abstract

Congenital anomalies of the coronary sinus and veins have been well documented, but only one instance of an anomalous small cardiac vein draining into the superior vena cava (SVC) has been reported. The majority of patients with anomalies of the coronary venous system are asymptomatic, but these variants are important to document as they may have clinically significant implications in the management and possible interventions patients may receive. This report describes an anomalous connection from the coronary venous system to the superior vena cava discovered incidentally in a patient with SVC syndrome and end-stage renal disease (ESRD). This may reflect a congenital variant which accommodated collateral flow to bypass the fully occluded SVC. Alternatively, it may be the result of repeated venoplasty of the stenotic SVC which opened an iatrogenic tract that was maintained and vascularized over time.

## Introduction

The small cardiac vein is found in 30–50% of patients and drains the right side of the heart. It is classically described as a tributary of the coronary sinus (85%), but can drain directly into the middle cardiac vein (12%), or directly into the right atrium (1%) [1]. This vein is a small diameter vessel, usually 1 mm or less, which originates near the inferior interventricular groove and ascends along the right ventricle into the right coronary sinus. It receives blood from the right atrium and diaphragmatic surface of the right ventricle [2]. This case report describes a dilated (3–5 mm) small cardiac vein with an anomalous connection to the superior vena cava. The incidence of this anomaly and the clinical implications are not known. A literature search reveals only one instance of this anomaly previously documented [3].

Patients with end-stage renal disease (ESRD) on dialysis who are treated with long-term indwelling central venous catheters are known to develop stenosis of the great vessels. Without a surgical arteriovenous fistula (AVF), access for life-sustaining hemodialysis is provided through an implanted foreign body. The tip of this hemodialysis catheter must be positioned in a large caliber vessel such as the superior vena cava (SVC) or inferior vena cava (IVC) to ensure adequate flow. Scar formation with possible stricture of the central venous circulation is a known consequence of repeated access and long-term instrumentation [4]. In severe instances, SVC syndrome may develop, where the SVC becomes partially or completely occluded due to intravascular scarring. In that setting, blood finds alternative routes to the heart, such as through the azygos system and collateralization through veins of the abdominal wall.

Known consequences of vascular injuries include the formation of arteriovenous (AV) fistulae, which may form over time from inadvertent puncture of an artery and a vein in close proximity [5]. The formation of a venovenous fistula is also possible if an iatrogenic tract is created and flow is maintained through that tract. Vasculogenesis is mediated by vascular endothelial growth factor at sites of injury which mobilizes endothelial progenitor cells from the bone marrow [6]. Blood will flow from a high-pressure area such as that found in an artery to a lower pressure area, such as a vein. In the case of SVC syndrome, the strictured SVC which accommodates all venous outflow from the head and upper extremities will have high intravascular pressure. This deoxygenated blood will flow down its pressure gradient through collaterals to reach its destination, the right heart.

## Case presentation

**Figure 1:**
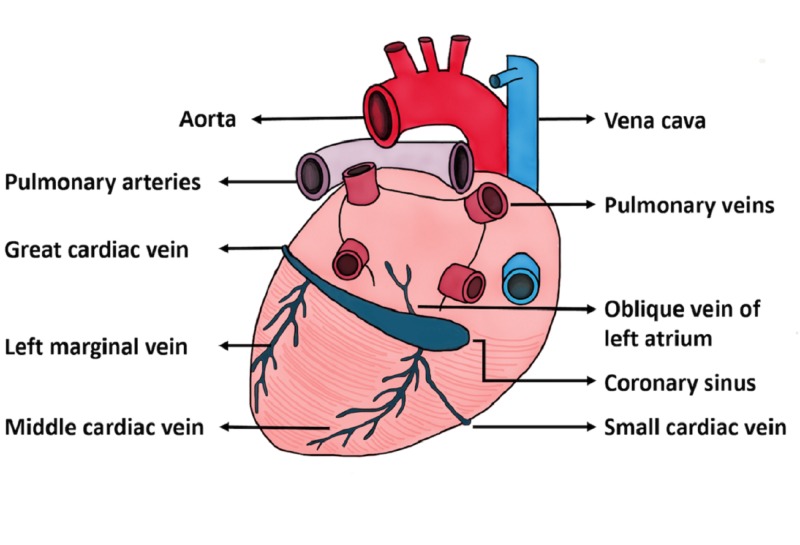
Illustration of Cardiac Vessel Anatomy. Artist rendering of normal venous cardiac anatomy.

The patient is a 39-year-old Caucasian male with long-standing ESRD who presented for evaluation and exchange of a malfunctioning internal jugular hemodialysis catheter. Notably there was chronic hemodialysis access malfunction due to known SVC stricture with four previous SVC venoplasties performed. The normal anatomic orientation of the cardiac vessels is depicted in Figure 1 for reference.

**Figure 2:**
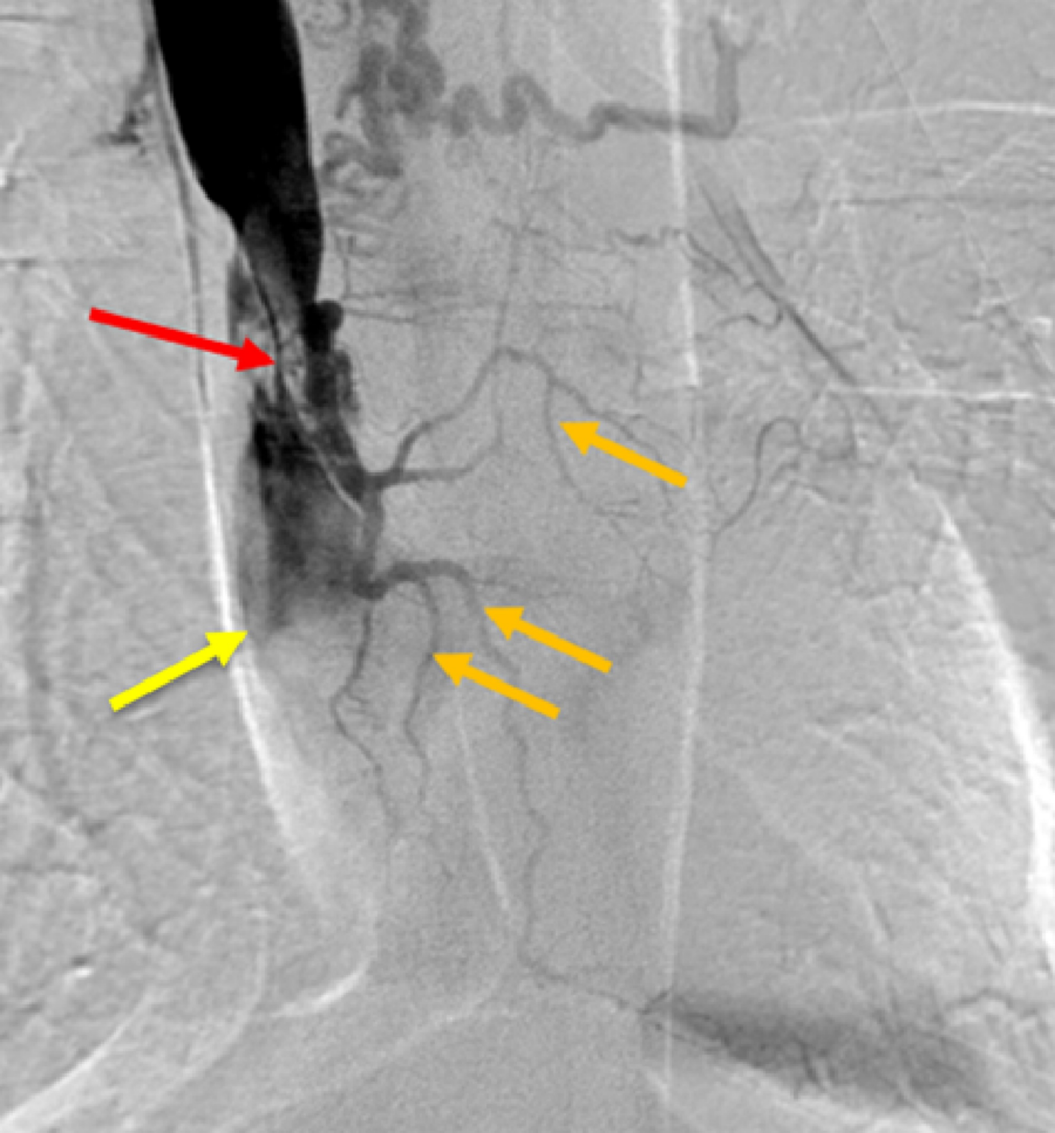
Occluded Superior Vena Cava (SVC). Central venogram of the SVC demonstrating stenosis of the superior vena cava (red arrow) and distal SVC total occlusion (yellow arrow). Multiple dilated collateral vessels are shown draping over the heart including the small cardiac vein and distal branches (orange arrows).

In this patient, an anomalous small cardiac vein draining directly into the SVC was incidentally discovered during evaluation and exchange of the malfunctioning tunneled hemodialysis catheter. Using manual dissection, the dialysis catheter was withdrawn, and a central venogram was performed to evaluate the proximal venous structures for possible stenosis or occlusion (Figure 2).

**Figure 3:**
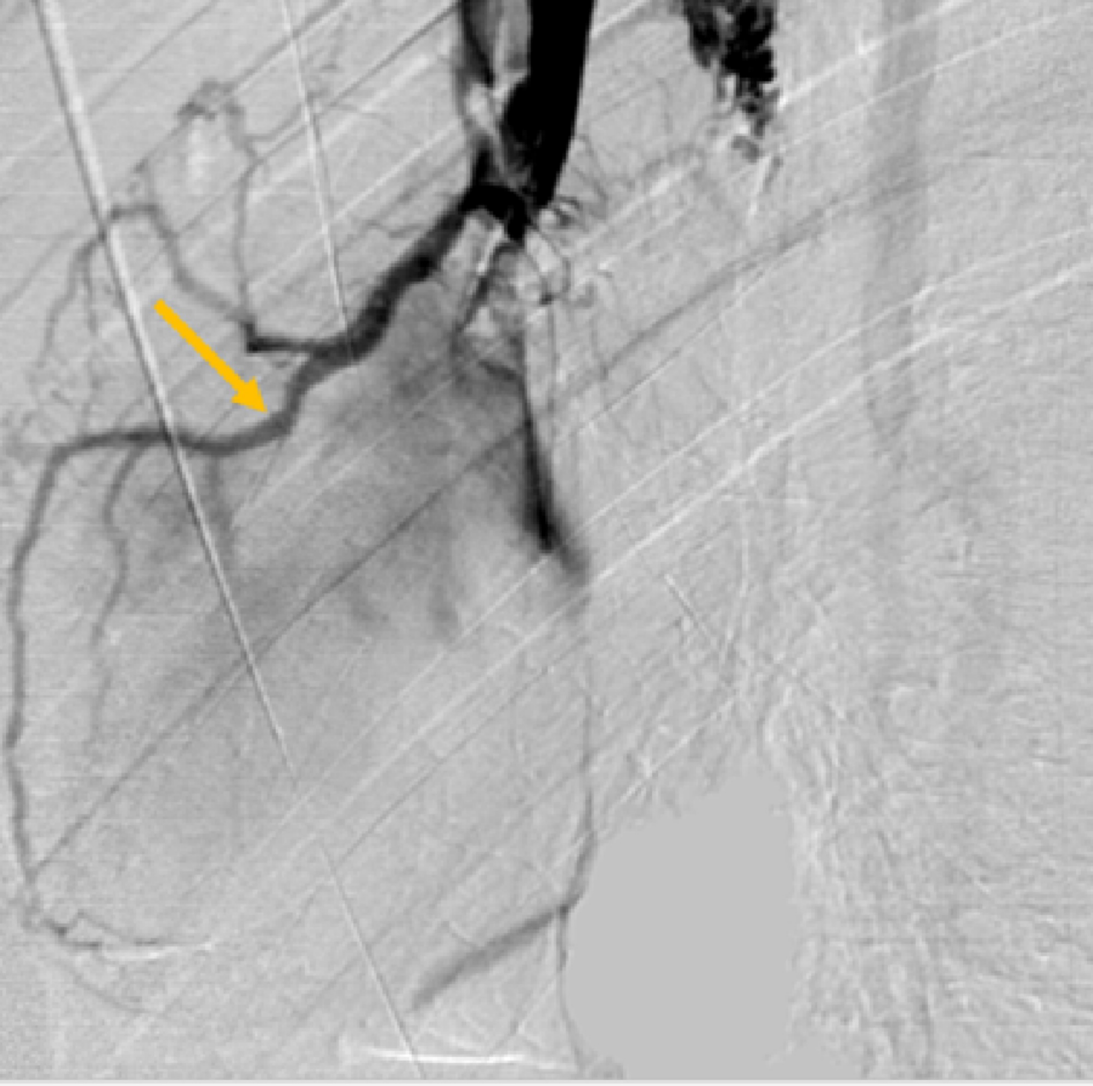
Venogram of Small Cardiac Vein. Repeat venogram with catheter slightly retracted demonstrates an anomalous small cardiac vein, as it drapes over the external border of the right heart (orange arrow).

High grade SVC stenosis was noted near the cavoatrial junction with filling of multiple collateral veins in the vicinity of the heart. Additional venograms were performed to further characterize this unusual anatomy (Figure 3).

**Figure 4:**
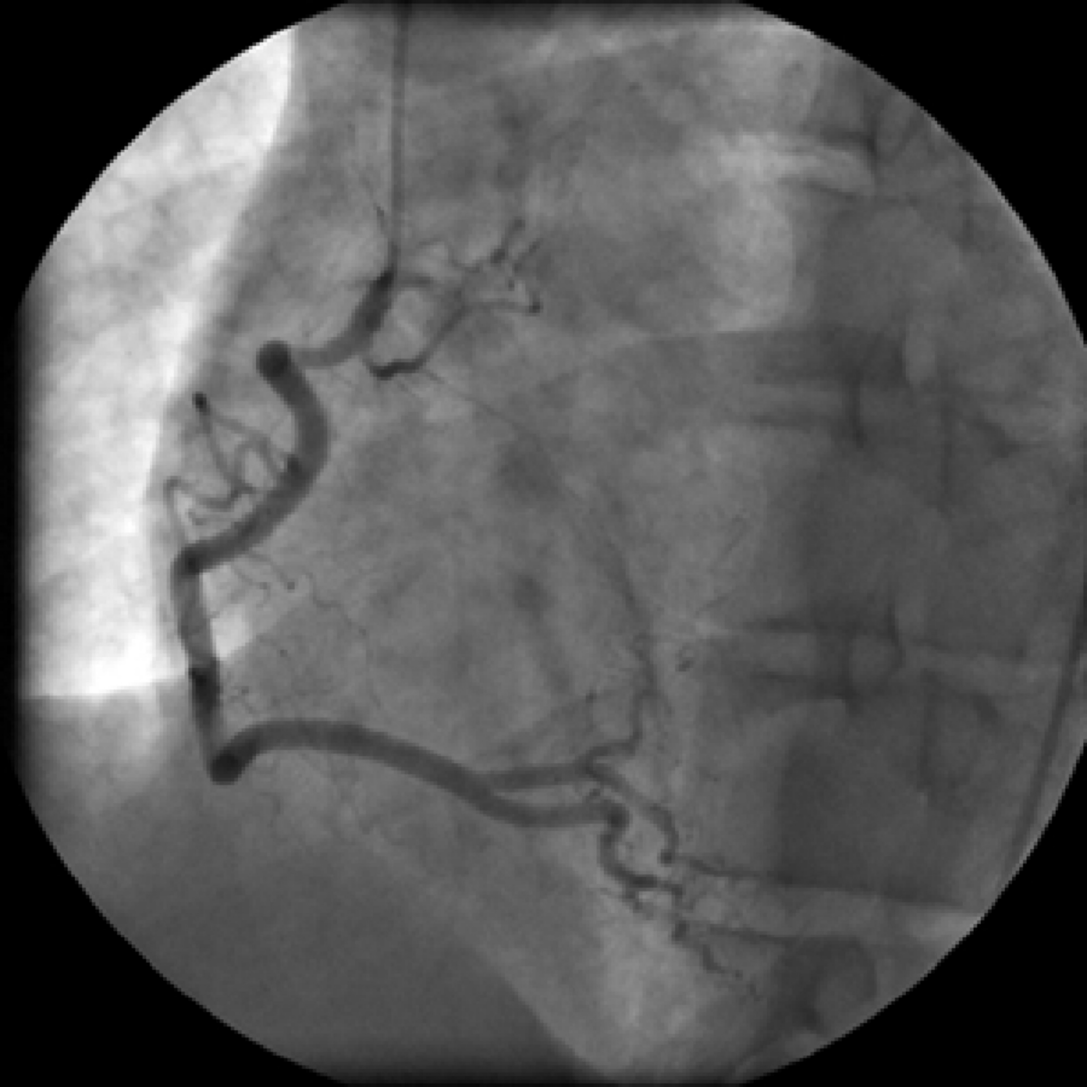
Right Coronary Angiogram. Selective right coronary angiography demonstrating normal anatomy, without evidence of abnormal communication with the superior vena cava (SVC).

These subsequent venograms demonstrated two vascular structures which followed the contour of the right heart border (small cardiac vein) and base of the heart (coronary sinus). Additionally, refluxed contrast was seen within the azygos vein, excluding this as a possible explanation for the abnormal vascular filling. The immediate and most pressing concern was a possible arteriovenous fistula involving the right coronary artery and the SVC. Cardiology was consulted and the patient was taken to the cardiology department for coronary arteriogram. Directed angiographic evaluation of the coronary arteries demonstrated no evidence of fistulous communication of the SVC with the coronary arterial system (Figure 4).

**Figure 5:**
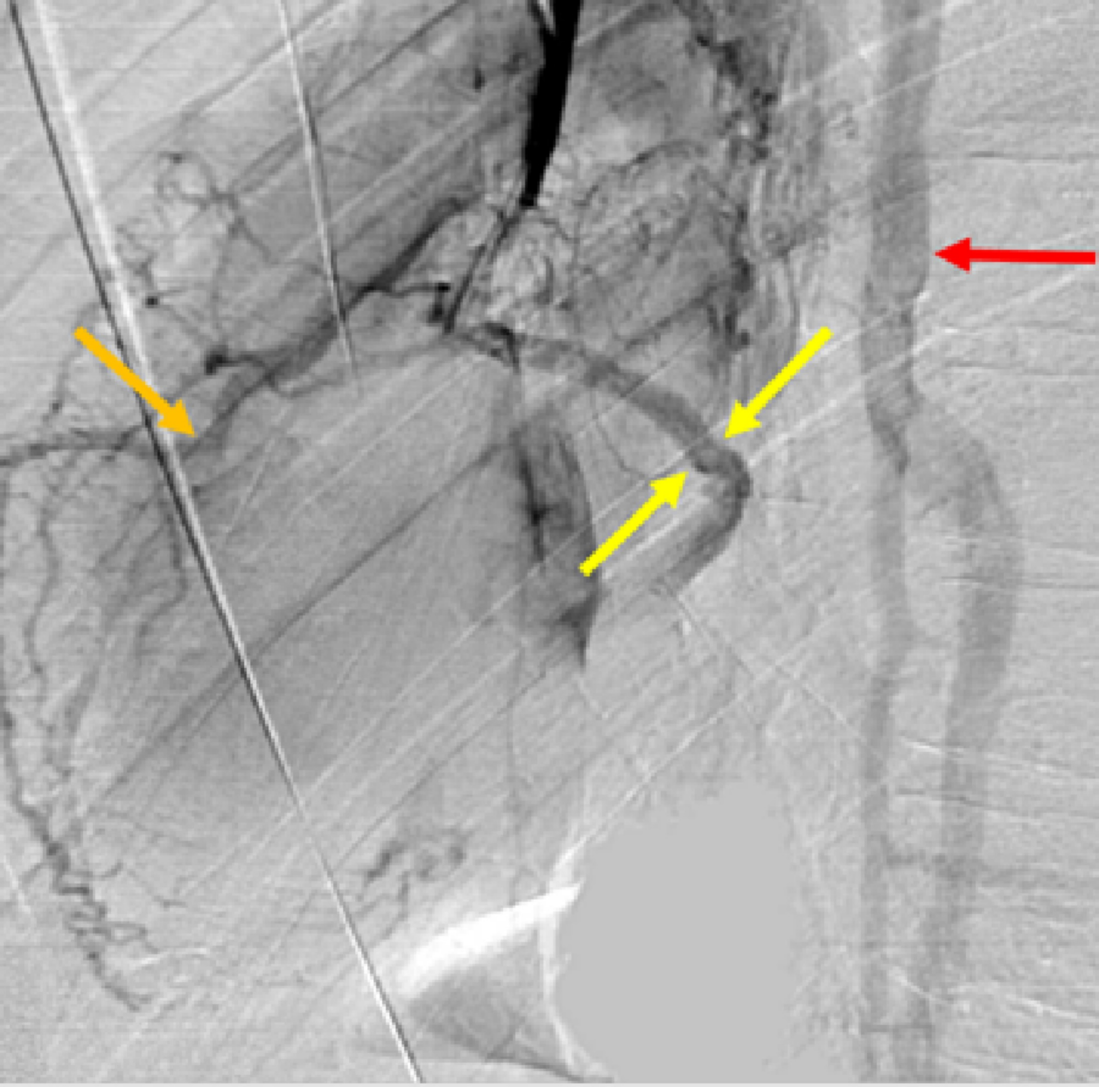
Cardiac Venogram of Small Cardiac Vein and Coronary Sinus. Delayed imaging venogram from a selective coronary artery angiogram shows the small cardiac vein (orange arrow); filling of the coronary sinus near the base of the heart (yellow arrows); filling of the azygos system as a result of refluxed contrast (red arrow).

Delayed views did show contrast enhancement of the previously identified abnormal vessel, confirming an anomalous venous drainage of the right heart. On repeat venogram, the small cardiac vein had direct communication with the superior vena cava and there was early filling of the coronary sinus (Figure 5).

Contrast enhancement of the coronary sinus did not occur when the catheter was slightly retracted and additional venogram was performed, likely indicating collateralization from the small cardiac vein. Notably, the small cardiac vein was significantly dilated measuring 3–5 mm, indicating a chronic process with dilation occurring due to worsening stenosis of the superior vena cava.

## Discussion

Optimal cardiac care is provided when clinicians have detailed knowledge of coronary anatomy, including its venous system. Clinical consideration has long been focused on the arterial coronary anatomy, but with improvement in cardiac imaging in recent years venous anomalies are being increasingly recognized. This is clinically significant as coronary venous interventions are becoming more prevalent including cardiac resynchronization therapy, mapping of arrhythmogenic foci and radiofrequency ablation, retrograde cardioplegia for both cardiac surgery and ischemic heart disease, and delivery of therapeutic agents [7]. In addition, these lesions may result in hemodynamically significant shunts and can be associated with congenital heart disease [8].

This patient may have had a congenital variant which was then accentuated by central venous stenosis due to multiple endovascular procedures. Alternatively, due to this high-grade stenosis an abnormal connection may have been iatrogenically created between the SVC and the coronary venous system. This would cause the small cardiac vein to dilate over time as a result of the increased flow from the systemic circulation of the head and neck. Pragmatically, this would bypass the strictured SVC, supplying deoxygenated blood directly to the right atrium through the coronary sinus.

While the patient was asymptomatic, discovery of the abnormal communication could prove to be clinically relevant. In the setting of long-standing ESRD, the patient’s life-sustaining SVC hemodialysis access may be precluded. Patients with ESRD frequently have complications involving their hemodialysis access that increase in frequency and severity with duration of treatment [9]. The incidence of this anatomic variant, the scope of potential clinical symptoms, and possible associated congenital abnormalities remain unknown and may be worthwhile areas of further research.

## Conclusions

This case demonstrates a rarely reported incidence of anomalous venous drainage with the small cardiac vein draining into the SVC. A thorough understanding of cardiac venous anatomy including potential variants is crucial when performing vascular procedures which may involve the venous system of the heart. Collateralization of the small cardiac vein may occur iatrogenically or in the presence of a congenital venous anomaly.
